# Laboratory Diagnosis of Antiphospholipid Antibodies

**DOI:** 10.1155/S1064744997000276

**Published:** 1997

**Authors:** M. M. Samama, M. H. Horellou, J. Conard

**Affiliations:** Service d'Hématologic Biologique (Pr J. P. Marie) Hotel-Diêu 1 place du Parvis Notre-Dame Paris Cedex 04 75181 France


Infectious Diseases in Obstetrics and Gynecology 5:181–182 (1997)

© 1997 Wiley-Liss, Inc.

### Laboratory Diagnosis of Antiphospholipid Antibodies

**M.M. Samama, M.H. Horellou, and J. Conard**

*Service d'Hématologic Biologique (Pr J. P. Marie), Hotel-Diêu, Paris, France*

Antiphospholipid antibodies may be associated with venous thromboembolism and/or arterial thromboses or miscarriages; their heterogeneity complicates the laboratory diagnosis. For a long time, they were believed to be directed against anionic phospholipids. More recently, it has been demonstrated that these antibodies are directed at plasma proteins bound most of the time to a phospholipid surface (anionic phospholipids). Several cardiolipin antibodies and lupus anticoagulant belong to this variety of antibodies. An extremely rich literature is available on this subject.[1–4]

The laboratory diagnosis of the so-called "Antiphospholipid Syndrome," is relatively difficult because of the heterogeneity of the antibodies, the large number of laboratory tests which have been advocated, and their standardization which has to be improved for many of them.[2]

Laboratory diagnosis is usually considered in patients with unexplained venous and/or arterial thromboses, recurrent miscarriages, and thrombocytopenia.[5,6] However, in several subjects the detection of a lupus or lupus-like circulating anticoagulant is made during preoperative laboratory testing, particularly in a child before tonsillectomy. The clinical significance of these latter antibodies is different than that of the previous group of symptomatic patients. The measurement of anticardiolipin (aCL) antibodies should be performed in parallel in every patient with a suspected antiphospholipid syndrome. Many recommendations concerning the preanalytical variables, especially regarding the risk of platelet contamination which may suppress the anticoagulant activity, must be taken into account.[2,4,7] The laboratory diagnosis of a lupus anticoagulant (LAC) relies on a four-step procedure for blood examination.

1. In the first step, the prolongation of the partial thromboplastin time with a ratio patient/control superior to 1:20 is detected. Several reagents are available, and the selection of a sensitive test is crucial.[7]
2. In the second step, it is essential to demonstrate that the addition of patient's plasma to a control plasma prolongs the activated partial thromboplastin time of the latter. In few cases the clotting time is longer than that of the patient. The measurement of the APTT of the control plasma, the patient's plasma, and their mixture (one volume of each) leads to the calculation of the Rosner index, the normal values of which are lower than 13 or 15, according to different investigators.
3. In the third step, a laboratory test is selected in order to demonstrate the antiphospholipid dependence of the LAC. Several reagents have been proposed: phospholipids present in a platelet preparation, hexagonal phospholipids, dilute tissue thromboplastin reagent, or dilute Russel viper venom. Their respective sensitivity and specificity are matters of debate. A new screening test using Textarin venom seems to be very sensitive to LAC.
4. In the fourth step, a confirmatory test can be useful in order to demonstrate that a deficiency in factor VIII or factor II is not associated with LAC or in very rare cases falsely diagnosed as a lupus anticoagulant. This differential diagnosis is essential since inhibitors specifically directed at factor VIII or factor II are associated with severe hemorrhages in contrast to the LAC.

The differential diagnosis of LAC and factor VIII antibody relies on the measurement of plasma factor VIII concentration in increasing dilutions of the patient plasma (1 to 10, 1 to 20, and 1 to 40). In LAC the level of factor VIII increases when greater dilutions are used. In the presence of anti-factor

Address correspondence to: M.M. Samama, Hotel-Diêu, 1 place du Parvis Notre-Dame, 75181 Paris Cedex 04, France.
Received 1 October 1997
Accepted 21 October 1997

VIII, the factor VIII concentration remains constant when different plasma dilutions are used.

In patients with recurrent fetal losses, the laboratory diagnosis of LAC should be performed with very sensitive reagents and anticardiolipin antibodies measurement should be done in parallel. A solid phase ELISA method is used to measure aCL antibodies. IgG and IgM antibodies directed against phospholipids binding plasma proteins, β2 glycoprotein 1 or prothrombin essentially or directed at these glycoproteins themselves have been observed during the phospholipid antibody syndrome.[8,9] It is important to demonstrate that they persist at two consecutive tests with a 2 to 3 month interval.

Other glycoproteins associated with phospholipids are annexin V, high and low molecular weight kininogen, protein C, and protein S.

Since β2 GP1 is a required cofactor for anticardiolipin antibody binding in the ELISA,[9] four hypotheses have been proposed to explain the specificity of aCL:

1. CL is directly recognized by aCL.
2. β2 GP1-CL complex is the structure recognized by aCL.
3. β2 GP1 is the target antigen for aCL but the epitope is cryptic in the absence of CL.
4. The actual epitope for aCL appears on the native structure of β2 GP1.

Alloantibodies are usually directed at phospholipids and give a negative reaction for antibodies directed to β21 GP1. They are observed in patients with syphilis or with a viral infection including VIH.[10] In contrast, autoantibodies that are encountered in patients with venous and/or arterial thrombosis and/or recurrent fetal losses are frequently directed against β2 GP1 associated in some cases with cardiolipins. Very recently a relationship between the presence of antibodies of the IgM subtype directed at β2 GP1 and recurrent fetal losses has been reported.[6]

Thus, important progress in the classification of antiphospholipid antibodies and their laboratory detection has been achieved. This will lead to a better understanding of the clinical relevance of the presence in blood of these heterogeneous antibodies.[8] In patients with recurrent fetal losses it will be interesting to investigate in further studies whether IgM antibodies directed at β2 GP1 are related to the pathogenesis of recurrent abortions.

### REFERENCES

1. Carreras LO, Perastiero RR. Pathogenic role of antiprotein-phospholipid antibodies. Haemostasis 26:340–357, 1996.
2. Piette JC, Papo T, Amoura Z, Boffa MC. Anticorps antiphospholipides/co-facteurs. Qui son-tils? Pourquoi, quand et comment les rechercher? Justifient ils un traitement? Ann Med Intern 147:492–497, 1996.
3. Nahum-Goldberg S, Conti-Kelley AM, Greco TP. A family study of anticardiolipin antibodies and associated clinical conditions. Am J Med 98:473, 1995.
4. Arvieux J, Darnige I, Sarrot-Reymauld F. Les nouvelles cibles des anticorps "antiphospholipides." Rev Med Interne 18:292–302, 1997.
5. Ginsberg JS, Wells PS, Brill-Edwards P, et al. Antiphospholipid antibodies and venous thromboembolism. Blood 86:3685–3691, 1995.
6. Ferastiero RR, Martinuzzo, Cerrato, et al. Relationship of anti-β2-glycoprotein 1 and antiprothrombin antibodies to thrombosis and pregnancy loss in patients with antiphospholipid antibodies. Thromb Haemost 78: 1008–1014, 1997.
7. Triplett DA. Antiphospholipid protein antibodies. Laboratory detection and clinical relevance. Thromb Res 78:1–31, 1995.
8. d'Angelo A, Safa O, Crippa L, et al. Relationship of lupus anticoagulant, anticardiolipin, anti-β2-GP1 and antiprothrombin autoantibodies with history of thrombosis in patients with the clinical suspicion of APA-syndrome. Thromb Haemost 77:967–968 (letter), 1997.
9. Marsuura E, Ogarashi Y, Nagae II, et al. Anticardiolipin antibodies recognize β2 glycoprotein 1 structure altered by interacting with an oxygen modified solid phase surface. J Exp Med 179:457–462, 1994.
10. Pierangell SS, Goldsmith GH, Kenic S, Nigel Harris E. Differences in functional activity of anticardiolipin antibodies from patients with syphilis and those with antiphopholipid syndrome. Infection and Immunity 62: 4081–4084, 1994.
